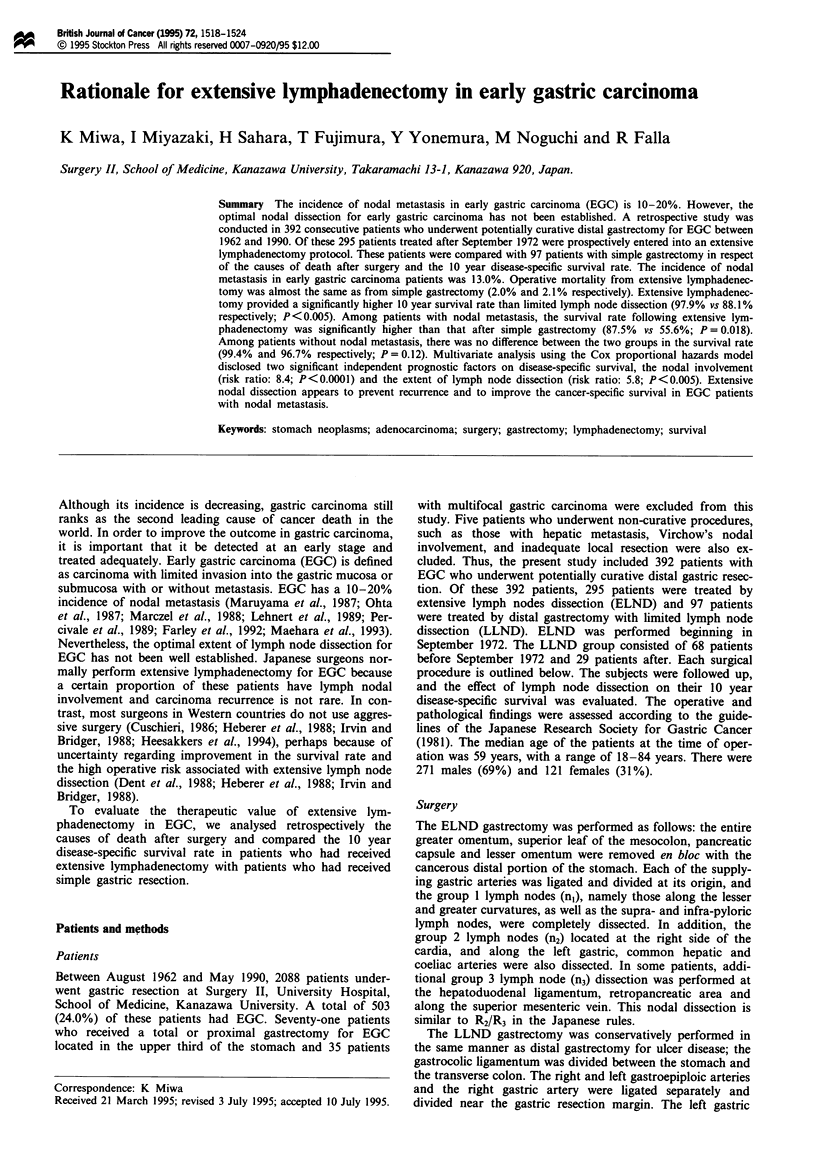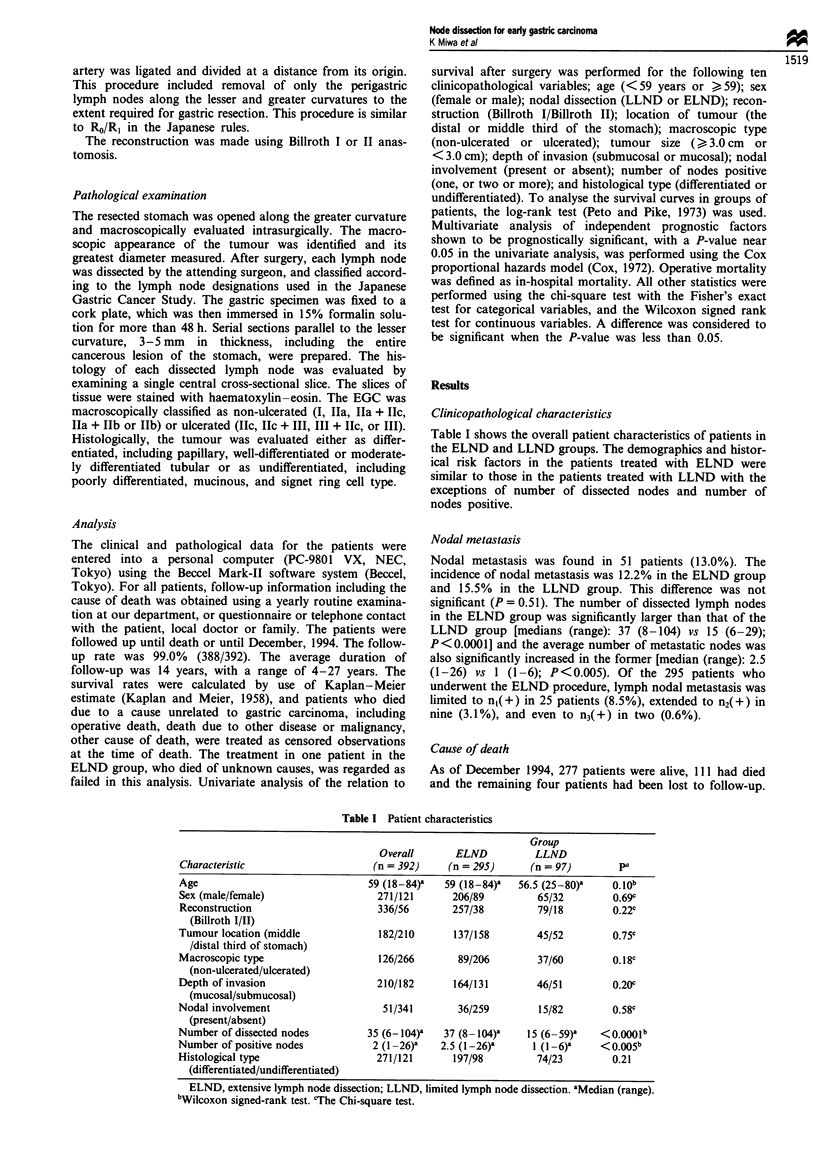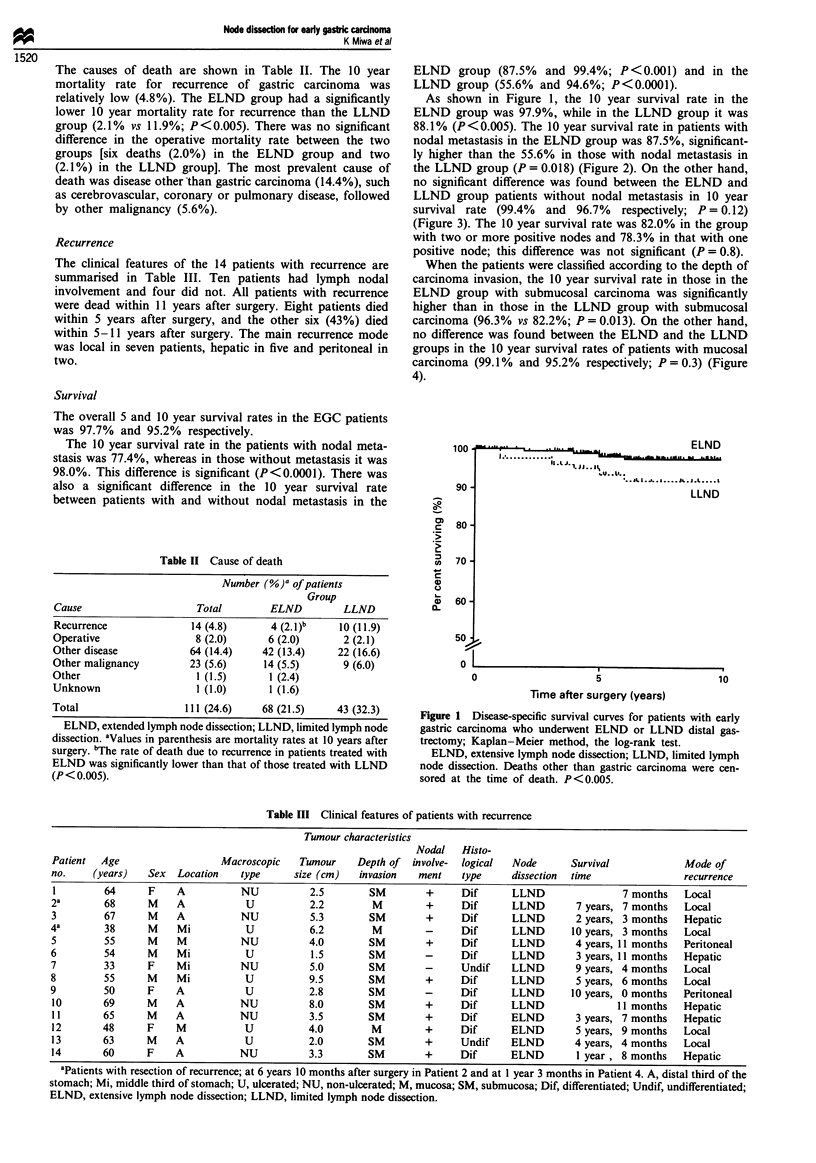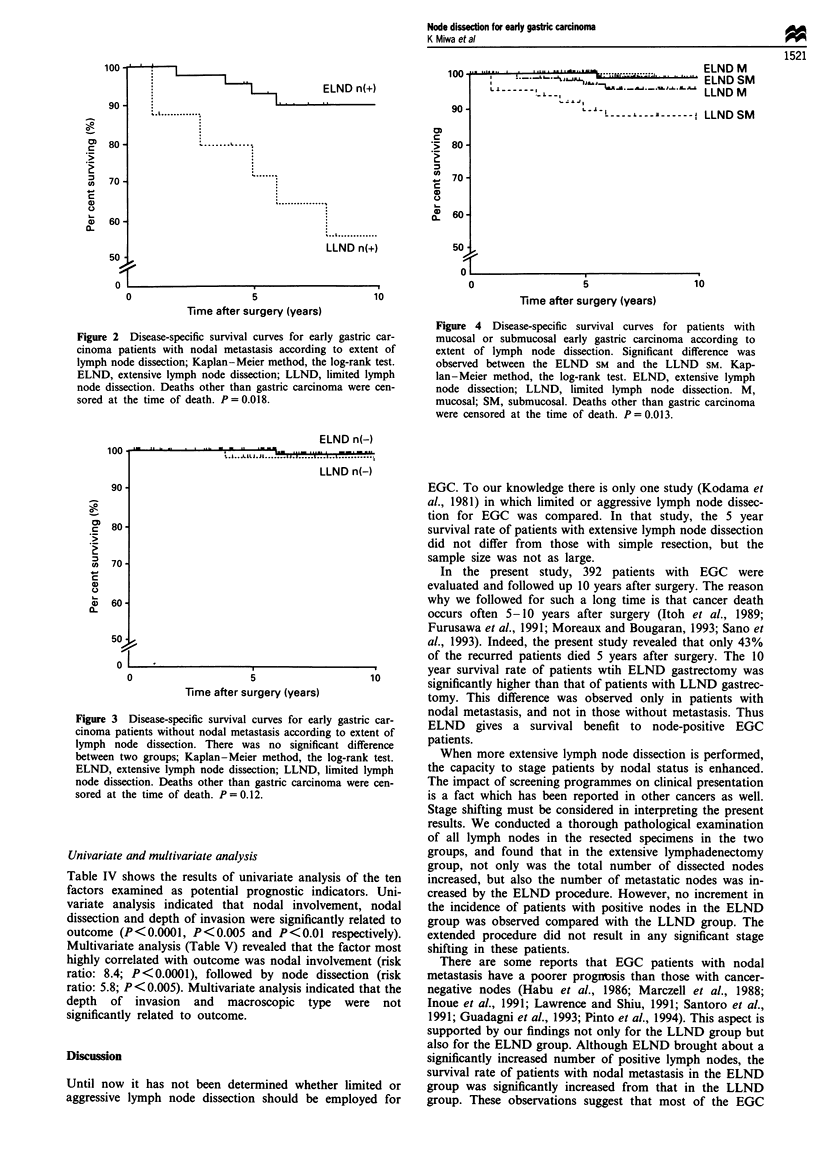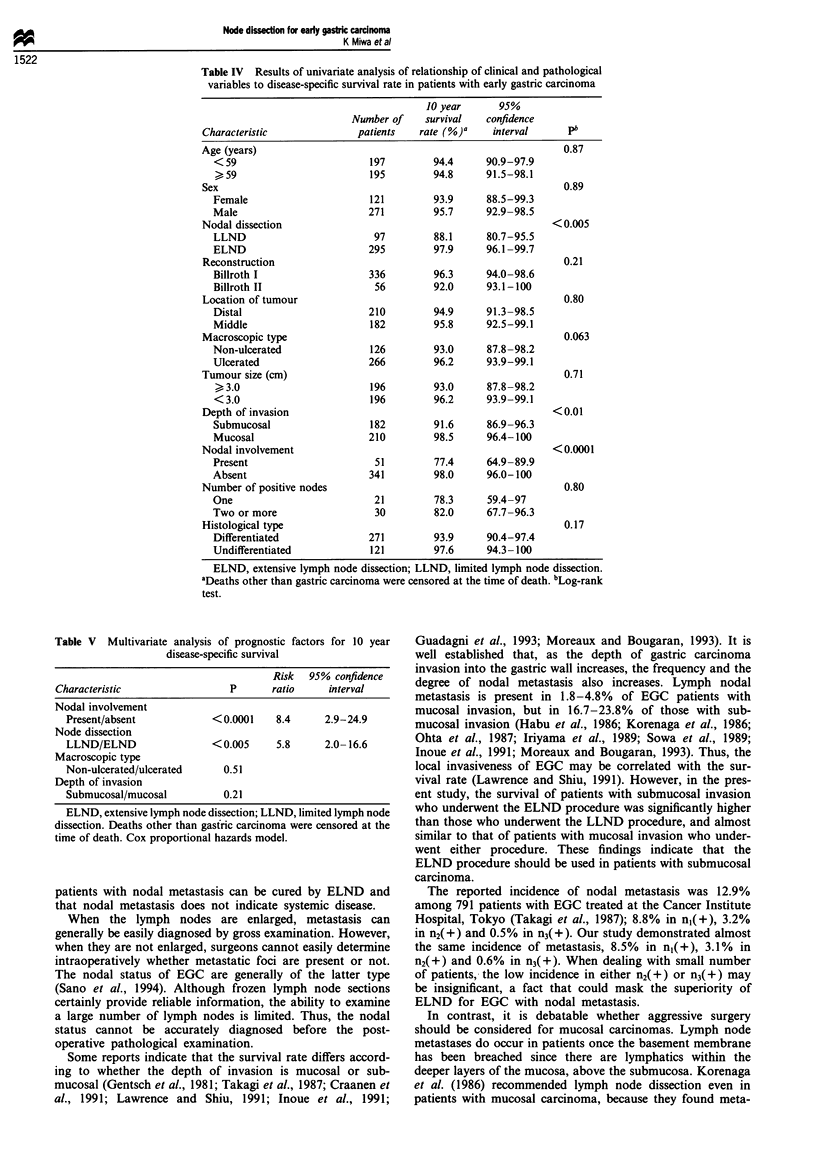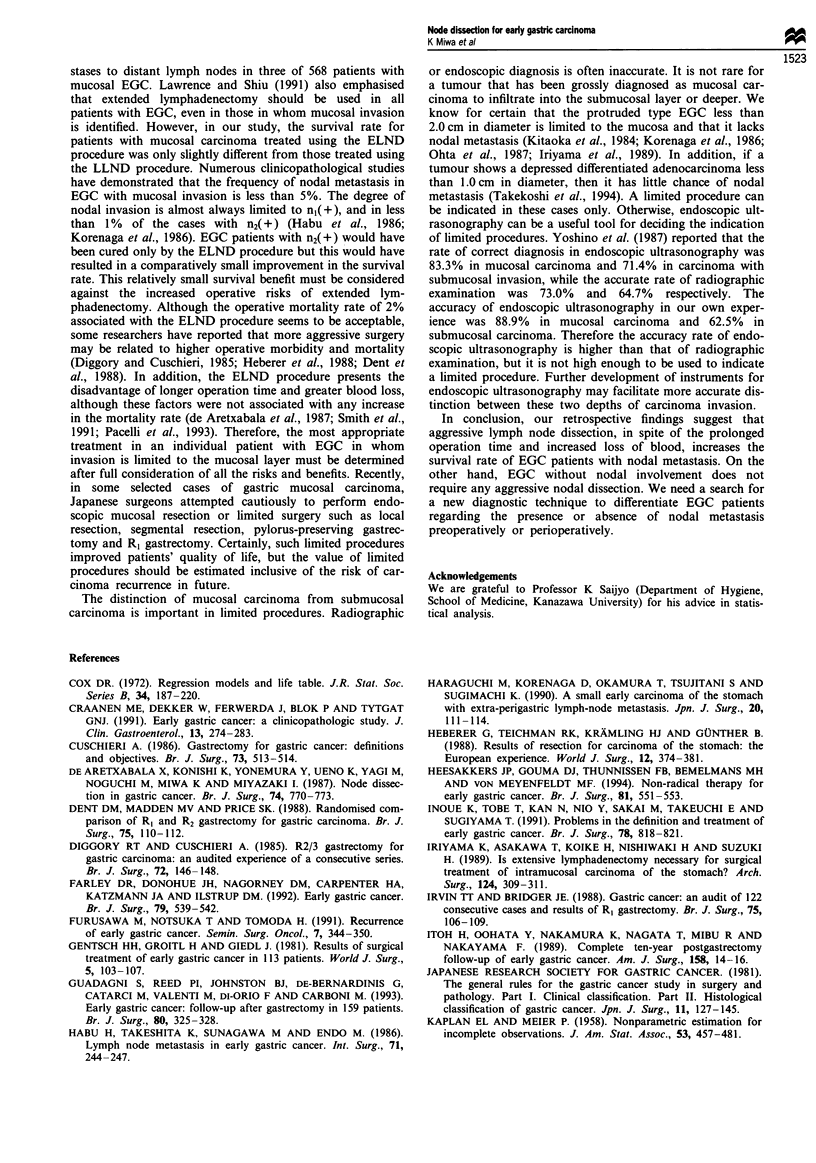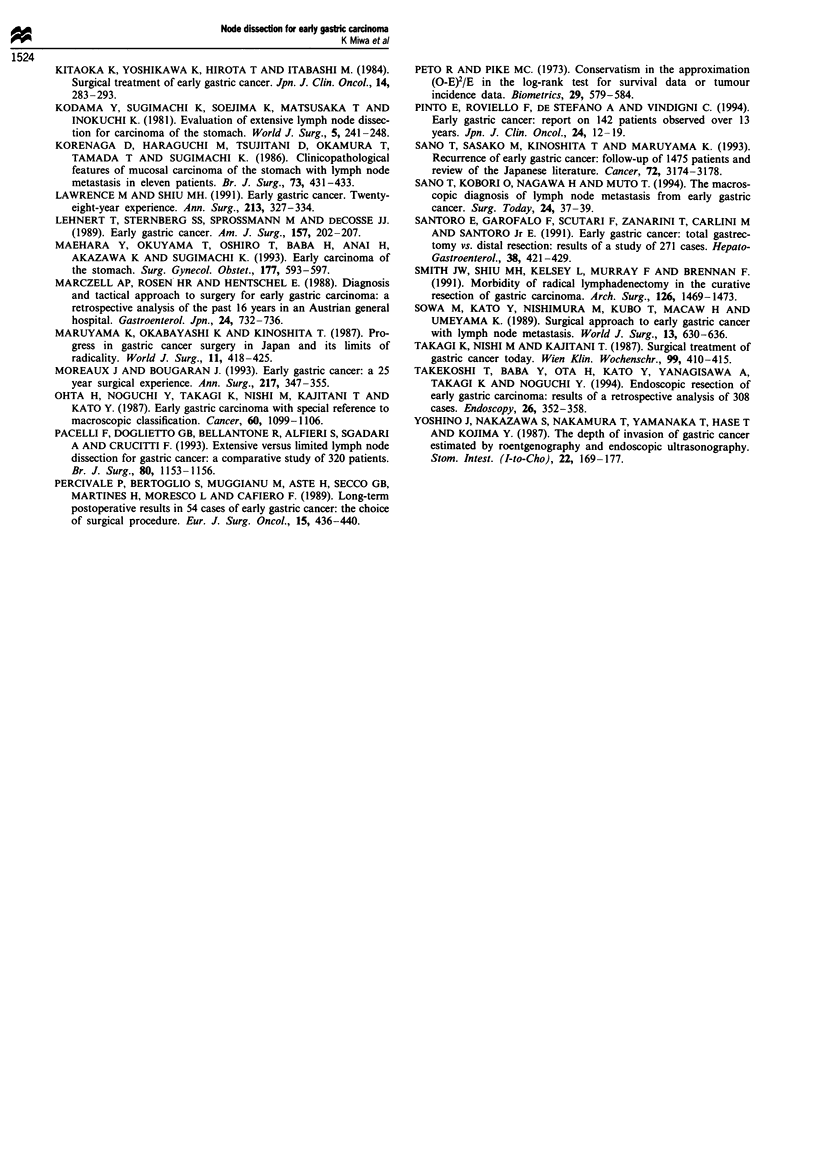# Rationale for extensive lymphadenectomy in early gastric carcinoma.

**DOI:** 10.1038/bjc.1995.540

**Published:** 1995-12

**Authors:** K. Miwa, I. Miyazaki, H. Sahara, T. Fujimura, Y. Yonemura, M. Noguchi, R. Falla

**Affiliations:** Surgery II, School of Medicine, Kanazawa University, Japan.

## Abstract

The incidence of nodal metastasis in early gastric carcinoma (EGC) is 10-20%. However, the optimal nodal dissection for early gastric carcinoma has not been established. A retrospective study was conducted in 392 consecutive patients who underwent potentially curative distal gastrectomy for EGC between 1962 and 1990. Of these 295 patients treated after September 1972 were prospectively entered into an extensive lymphadenectomy protocol. These patients were compared with 97 patients with simple gastrectomy in respect of the causes of death after surgery and the 10 year disease-specific survival rate. The incidence of nodal metastasis in early gastric carcinoma patients was 13.0%. Operative mortality from extensive lymphadenectomy was almost the same as from simple gastrectomy (2.0% and 2.1% respectively). Extensive lymphadenectomy provided a significantly higher 10 year survival rate than limited lymph node dissection (97.9% vs 88.1% respectively; P < 0.005). Among patients with nodal metastasis, the survival rate following extensive lymphadenectomy was significantly higher than that after simple gastrectomy (87.5% vs 55.6%; P = 0.018). Among patients without nodal metastasis, there was no difference between the two groups in the survival rate (99.4% and 96.7% respectively; P = 0.12). Multivariate analysis using the Cox proportional hazards model disclosed two significant independent prognostic factors on disease-specific survival, the nodal involvement (risk ratio: 8.4; P < 0.0001) and the extent of lymph node dissection (risk ratio: 5.8; P < 0.005). Extensive nodel dissection appears to prevent recurrence and to improve the cancer-specific survival in EGC patients with nodal metastasis.


British Journal of Cancer (1995) 72, 1518-1524
© 1995 Stockton Press All rights reserved 0007-0920/95 $12.00

### Rationale for extensive lymphadenectomy in early gastric carcinoma

K Miwa, I Miyazaki, H Sahara, T Fujimura, Y Yonemura, M Noguchi and R Falla

*Surgery II, School of Medicine, Kanazawa University, Takaramachi 13-1, Kanazawa 920, Japan.*

**Summary** The incidence of nodal metastasis in early gastric carcinoma (EGC) is 10-20%. However, the optimal nodal dissection for early gastric carcinoma has not been established. A retrospective study was conducted in 392 consecutive patients who underwent potentially curative distal gastrectomy for EGC between 1962 and 1990. Of these 295 patients treated after September 1972 were prospectively entered into an extensive lymphadenectomy protocol. These patients were compared with 97 patients with simple gastrectomy in respect of the causes of death after surgery and the 10 year disease-specific survival rate. The incidence of nodal metastasis in early gastric carcinoma patients was 13.0%. Operative mortality from extensive lymphadenectomy was almost the same as from simple gastrectomy (2.0% and 2.1% respectively). Extensive lymphadenectomy provided a significantly higher 10 year survival rate than limited lymph node dissection (97.9% *vs* 88.1% respectively; $P < 0.005$). Among patients with nodal metastasis, the survival rate following extensive lymphadenectomy was significantly higher than that after simple gastrectomy (87.5% *vs* 55.6%; $P = 0.018$). Among patients without nodal metastasis, there was no difference between the two groups in the survival rate (99.4% and 96.7% respectively; $P = 0.12$). Multivariate analysis using the Cox proportional hazards model disclosed two significant independent prognostic factors on disease-specific survival, the nodal involvement (risk ratio: 8.4; $P < 0.0001$) and the extent of lymph node dissection (risk ratio: 5.8; $P < 0.005$). Extensive nodal dissection appears to prevent recurrence and to improve the cancer-specific survival in EGC patients with nodal metastasis.

**Keywords**: stomach neoplasms; adenocarcinoma; surgery; gastrectomy; lymphadenectomy; survival

Although its incidence is decreasing, gastric carcinoma still ranks as the second leading cause of cancer death in the world. In order to improve the outcome in gastric carcinoma, it is important that it be detected at an early stage and treated adequately. Early gastric carcinoma (EGC) is defined as carcinoma with limited invasion into the gastric mucosa or submucosa with or without metastasis. EGC has a 10-20% incidence of nodal metastasis (Maruyama *et al.*, 1987; Ohta *et al.*, 1987; Marczel *et al.*, 1988; Lehnert *et al.*, 1989; Percivale *et al.*, 1989; Farley *et al.*, 1992; Maehara *et al.*, 1993). Nevertheless, the optimal extent of lymph node dissection for EGC has not been well established. Japanese surgeons normally perform extensive lymphadenectomy for EGC because a certain proportion of these patients have lymph nodal involvement and carcinoma recurrence is not rare. In contrast, most surgeons in Western countries do not use aggressive surgery (Cuschieri, 1986; Heberer *et al.*, 1988; Irvin and Bridger, 1988; Heesakkers *et al.*, 1994), perhaps because of uncertainty regarding improvement in the survival rate and the high operative risk associated with extensive lymph node dissection (Dent *et al.*, 1988; Heberer *et al.*, 1988; Irvin and Bridger, 1988).

To evaluate the therapeutic value of extensive lymphadenectomy in EGC, we analysed retrospectively the causes of death after surgery and compared the 10 year disease-specific survival rate in patients who had received extensive lymphadenectomy with patients who had received simple gastric resection.

### Patients and methods

### *Patients*

Between August 1962 and May 1990, 2088 patients underwent gastric resection at Surgery II, University Hospital, School of Medicine, Kanazawa University. A total of 503 (24.0%) of these patients had EGC. Seventy-one patients who received a total or proximal gastrectomy for EGC located in the upper third of the stomach and 35 patients with multifocal gastric carcinoma were excluded from this study. Five patients who underwent non-curative procedures, such as those with hepatic metastasis, Virchow's nodal involvement, and inadequate local resection were also excluded. Thus, the present study included 392 patients with EGC who underwent potentially curative distal gastric resection. Of these 392 patients, 295 patients were treated by extensive lymph nodes dissection (ELND) and 97 patients were treated by distal gastrectomy with limited lymph node dissection (LLND). ELND was performed beginning in September 1972. The LLND group consisted of 68 patients before September 1972 and 29 patients after. Each surgical procedure is outlined below. The subjects were followed up, and the effect of lymph node dissection on their 10 year disease-specific survival was evaluated. The operative and pathological findings were assessed according to the guidelines of the Japanese Research Society for Gastric Cancer (1981). The median age of the patients at the time of operation was 59 years, with a range of 18-84 years. There were 271 males (69%) and 121 females (31%).

### *Surgery*

The ELND gastrectomy was performed as follows: the entire greater omentum, superior leaf of the mesocolon, pancreatic capsule and lesser omentum were removed *en bloc* with the cancerous distal portion of the stomach. Each of the supplying gastric arteries was ligated and divided at its origin, and the group 1 lymph nodes ($n_1$), namely those along the lesser and greater curvatures, as well as the supra- and infra-pyloric lymph nodes, were completely dissected. In addition, the group 2 lymph nodes ($n_2$) located at the right side of the cardia, and along the left gastric, common hepatic and coeliac arteries were also dissected. In some patients, additional group 3 lymph node ($n_3$) dissection was performed at the hepatoduodenal ligamentum, retropancreatic area and along the superior mesenteric vein. This nodal dissection is similar to $R_2/R_3$ in the Japanese rules.

The LLND gastrectomy was conservatively performed in the same manner as distal gastrectomy for ulcer disease; the gastrocolic ligamentum was divided between the stomach and the transverse colon. The right and left gastroepiploic arteries and the right gastric artery were ligated separately and divided near the gastric resection margin. The left gastric

Correspondence: K Miwa
Received 21 March 1995; revised 3 July 1995; accepted 10 July 1995.

artery was ligated and divided at a distance from its origin. This procedure included removal of only the perigastric lymph nodes along the lesser and greater curvatures to the extent required for gastric resection. This procedure is similar to $R_0/R_1$ in the Japanese rules.

The reconstruction was made using Billroth I or II anastomosis.

### *Pathological examination*

The resected stomach was opened along the greater curvature and macroscopically evaluated intrasurgically. The macroscopic appearance of the tumour was identified and its greatest diameter measured. After surgery, each lymph node was dissected by the attending surgeon, and classified according to the lymph node designations used in the Japanese Gastric Cancer Study. The gastric specimen was fixed to a cork plate, which was then immersed in 15% formalin solution for more than 48 h. Serial sections parallel to the lesser curvature, 3–5 mm in thickness, including the entire cancerous lesion of the stomach, were prepared. The histology of each dissected lymph node was evaluated by examining a single central cross-sectional slice. The slices of tissue were stained with haematoxylin–eosin. The EGC was macroscopically classified as non-ulcerated (I, IIa, IIa + IIc, IIa + IIb or IIb) or ulcerated (IIc, IIc + III, III + IIc, or III). Histologically, the tumour was evaluated either as differentiated, including papillary, well-differentiated or moderately differentiated tubular or as undifferentiated, including poorly differentiated, mucinous, and signet ring cell type.

### *Analysis*

The clinical and pathological data for the patients were entered into a personal computer (PC-9801 VX, NEC, Tokyo) using the Beccel Mark-II software system (Beccel, Tokyo). For all patients, follow-up information including the cause of death was obtained using a yearly routine examination at our department, or questionnaire or telephone contact with the patient, local doctor or family. The patients were followed up until death or until December, 1994. The follow-up rate was 99.0% (388/392). The average duration of follow-up was 14 years, with a range of 4–27 years. The survival rates were calculated by use of Kaplan–Meier estimate (Kaplan and Meier, 1958), and patients who died due to a cause unrelated to gastric carcinoma, including operative death, death due to other disease or malignancy, other cause of death, were treated as censored observations at the time of death. The treatment in one patient in the ELND group, who died of unknown causes, was regarded as failed in this analysis. Univariate analysis of the relation to survival after surgery was performed for the following ten clinicopathological variables; age (<59 years or ⩾59); sex (female or male); nodal dissection (LLND or ELND); reconstruction (Billroth I/Billroth II); location of tumour (the distal or middle third of the stomach); macroscopic type (non-ulcerated or ulcerated); tumour size (⩾3.0 cm or <3.0 cm); depth of invasion (submucosal or mucosal); nodal involvement (present or absent); number of nodes positive (one, or two or more); and histological type (differentiated or undifferentiated). To analyse the survival curves in groups of patients, the log-rank test (Peto and Pike, 1973) was used. Multivariate analysis of independent prognostic factors shown to be prognostically significant, with a *P*-value near 0.05 in the univariate analysis, was performed using the Cox proportional hazards model (Cox, 1972). Operative mortality was defined as in-hospital mortality. All other statistics were performed using the chi-square test with the Fisher's exact test for categorical variables, and the Wilcoxon signed rank test for continuous variables. A difference was considered to be significant when the *P*-value was less than 0.05.

### Results

### *Clinicopathological characteristics*

Table I shows the overall patient characteristics of patients in the ELND and LLND groups. The demographics and historical risk factors in the patients treated with ELND were similar to those in the patients treated with LLND with the exceptions of number of dissected nodes and number of nodes positive.

### *Nodal metastasis*

Nodal metastasis was found in 51 patients (13.0%). The incidence of nodal metastasis was 12.2% in the ELND group and 15.5% in the LLND group. This difference was not significant ($P = 0.51$). The number of dissected lymph nodes in the ELND group was significantly larger than that of the LLND group [medians (range): 37 (8–104) *vs* 15 (6–29); $P < 0.0001$] and the average number of metastatic nodes was also significantly increased in the former [median (range): 2.5 (1–26) *vs* 1 (1–6); $P < 0.005$). Of the 295 patients who underwent the ELND procedure, lymph nodal metastasis was limited to $n_1(+)$ in 25 patients (8.5%), extended to $n_2(+)$ in nine (3.1%), and even to $n_3(+)$ in two (0.6%).

### *Cause of death*

As of December 1994, 277 patients were alive, 111 had died and the remaining four patients had been lost to follow-up.

**Table I** Patient characteristics

| *Characteristic* | *Overall* (n = 392) | *ELND* (n = 295) | *Group LLND* (n = 97) | P[a] |
|---|---|---|---|---|
| Age | 59 (18–84)[a] | 59 (18–84)[a] | 56.5 (25–80)[a] | 0.10[b] |
| Sex (male/female) | 271/121 | 206/89 | 65/32 | 0.69[c] |
| Reconstruction (Billroth I/II) | 336/56 | 257/38 | 79/18 | 0.22[c] |
| Tumour location (middle /distal third of stomach) | 182/210 | 137/158 | 45/52 | 0.75[c] |
| Macroscopic type (non-ulcerated/ulcerated) | 126/266 | 89/206 | 37/60 | 0.18[c] |
| Depth of invasion (mucosal/submucosal) | 210/182 | 164/131 | 46/51 | 0.20[c] |
| Nodal involvement (present/absent) | 51/341 | 36/259 | 15/82 | 0.58[c] |
| Number of dissected nodes | 35 (6–104)[a] | 37 (8–104)[a] | 15 (6–59)[a] | <0.0001[b] |
| Number of positive nodes | 2 (1–26)[a] | 2.5 (1–26)[a] | 1 (1–6)[a] | <0.005[b] |
| Histological type (differentiated/undifferentiated) | 271/121 | 197/98 | 74/23 | 0.21 |

ELND, extensive lymph node dissection; LLND, limited lymph node dissection. [a]Median (range). [b]Wilcoxon signed-rank test. [c]The Chi-square test.

The causes of death are shown in Table II. The 10 year mortality rate for recurrence of gastric carcinoma was relatively low (4.8%). The ELND group had a significantly lower 10 year mortality rate for recurrence than the LLND group (2.1% *vs* 11.9%; $P<0.005$). There was no significant difference in the operative mortality rate between the two groups [six deaths (2.0%) in the ELND group and two (2.1%) in the LLND group]. The most prevalent cause of death was disease other than gastric carcinoma (14.4%), such as cerebrovascular, coronary or pulmonary disease, followed by other malignancy (5.6%).

### *Recurrence*

The clinical features of the 14 patients with recurrence are summarised in Table III. Ten patients had lymph nodal involvement and four did not. All patients with recurrence were dead within 11 years after surgery. Eight patients died within 5 years after surgery, and the other six (43%) died within 5–11 years after surgery. The main recurrence mode was local in seven patients, hepatic in five and peritoneal in two.

### *Survival*

The overall 5 and 10 year survival rates in the EGC patients was 97.7% and 95.2% respectively.

The 10 year survival rate in the patients with nodal metastasis was 77.4%, whereas in those without metastasis it was 98.0%. This difference is significant ($P<0.0001$). There was also a significant difference in the 10 year survival rate between patients with and without nodal metastasis in the ELND group (87.5% and 99.4%; $P<0.001$) and in the LLND group (55.6% and 94.6%; $P<0.0001$).

As shown in Figure 1, the 10 year survival rate in the ELND group was 97.9%, while in the LLND group it was 88.1% ($P<0.005$). The 10 year survival rate in patients with nodal metastasis in the ELND group was 87.5%, significantly higher than the 55.6% in those with nodal metastasis in the LLND group ($P=0.018$) (Figure 2). On the other hand, no significant difference was found between the ELND and LLND group patients without nodal metastasis in 10 year survival rate (99.4% and 96.7% respectively; $P=0.12$) (Figure 3). The 10 year survival rate was 82.0% in the group with two or more positive nodes and 78.3% in that with one positive node; this difference was not significant ($P=0.8$).

When the patients were classified according to the depth of carcinoma invasion, the 10 year survival rate in those in the ELND group with submucosal carcinoma was significantly higher than in those in the LLND group with submucosal carcinoma (96.3% *vs* 82.2%; $P=0.013$). On the other hand, no difference was found between the ELND and the LLND groups in the 10 year survival rates of patients with mucosal carcinoma (99.1% and 95.2% respectively; $P=0.3$) (Figure 4).

**Table II** Cause of death

| *Cause* | *Number (%)[a] of patients* *Total* | *Group* *ELND* | *LLND* |
|---|---|---|---|
| Recurrence | 14 (4.8) | 4 (2.1)[b] | 10 (11.9) |
| Operative | 8 (2.0) | 6 (2.0) | 2 (2.1) |
| Other disease | 64 (14.4) | 42 (13.4) | 22 (16.6) |
| Other malignancy | 23 (5.6) | 14 (5.5) | 9 (6.0) |
| Other | 1 (1.5) | 1 (2.4) | |
| Unknown | 1 (1.0) | 1 (1.6) | |
| Total | 111 (24.6) | 68 (21.5) | 43 (32.3) |

ELND, extended lymph node dissection; LLND, limited lymph node dissection. [a]Values in parenthesis are mortality rates at 10 years after surgery. [b]The rate of death due to recurrence in patients treated with ELND was significantly lower than that of those treated with LLND ($P<0.005$).

**Figure 1** Disease-specific survival curves for patients with early gastric carcinoma who underwent ELND or LLND distal gastrectomy; Kaplan–Meier method, the log-rank test.

ELND, extensive lymph node dissection; LLND, limited lymph node dissection. Deaths other than gastric carcinoma were censored at the time of death. $P<0.005$.

**Table III** Clinical features of patients with recurrence

| *Patient no.* | *Age (years)* | *Sex* | *Tumour characteristics* *Location* | *Macroscopic type* | *Tumour size (cm)* | *Depth of invasion* | *Nodal involvement* | *Histological type* | *Node dissection* | *Survival time* | *Mode of recurrence* |
|---|---|---|---|---|---|---|---|---|---|---|---|
| 1 | 64 | F | A | NU | 2.5 | SM | + | Dif | LLND | 7 months | Local |
| 2[a] | 68 | M | A | U | 2.2 | M | + | Dif | LLND | 7 years, 7 months | Local |
| 3 | 67 | M | A | NU | 5.3 | SM | + | Dif | LLND | 2 years, 3 months | Hepatic |
| 4[a] | 38 | M | Mi | U | 6.2 | M | – | Dif | LLND | 10 years, 3 months | Local |
| 5 | 55 | M | M | NU | 4.0 | SM | + | Dif | LLND | 4 years, 11 months | Peritoneal |
| 6 | 54 | M | Mi | U | 1.5 | SM | – | Dif | LLND | 3 years, 11 months | Hepatic |
| 7 | 33 | F | Mi | NU | 5.0 | SM | – | Undif | LLND | 9 years, 4 months | Local |
| 8 | 55 | M | Mi | U | 9.5 | SM | + | Dif | LLND | 5 years, 6 months | Local |
| 9 | 50 | F | A | U | 2.8 | SM | – | Dif | LLND | 10 years, 0 months | Peritoneal |
| 10 | 69 | M | A | NU | 8.0 | SM | + | Dif | LLND | 11 months | Hepatic |
| 11 | 65 | M | A | NU | 3.5 | SM | + | Dif | ELND | 3 years, 7 months | Hepatic |
| 12 | 48 | F | M | U | 4.0 | M | + | Dif | ELND | 5 years, 9 months | Local |
| 13 | 63 | M | A | U | 2.0 | SM | + | Undif | ELND | 4 years, 4 months | Local |
| 14 | 60 | F | A | NU | 3.3 | SM | + | Dif | ELND | 1 year , 8 months | Hepatic |

[a]Patients with resection of recurrence; at 6 years 10 months after surgery in Patient 2 and at 1 year 3 months in Patient 4. A, distal third of the stomach; Mi, middle third of stomach; U, ulcerated; NU, non-ulcerated; M, mucosa; SM, submucosa; Dif, differentiated; Undif, undifferentiated; ELND, extensive lymph node dissection; LLND, limited lymph node dissection.

**Figure 2** Disease-specific survival curves for early gastric carcinoma patients with nodal metastasis according to extent of lymph node dissection; Kaplan–Meier method, the log-rank test. ELND, extensive lymph node dissection; LLND, limited lymph node dissection. Deaths other than gastric carcinoma were censored at the time of death. $P = 0.018$.

**Figure 3** Disease-specific survival curves for early gastric carcinoma patients without nodal metastasis according to extent of lymph node dissection. There was no significant difference between two groups; Kaplan–Meier method, the log-rank test. ELND, extensive lymph node dissection; LLND, limited lymph node dissection. Deaths other than gastric carcinoma were censored at the time of death. $P = 0.12$.

**Figure 4** Disease-specific survival curves for patients with mucosal or submucosal early gastric carcinoma according to extent of lymph node dissection. Significant difference was observed between the ELND SM and the LLND SM. Kaplan–Meier method, the log-rank test. ELND, extensive lymph node dissection; LLND, limited lymph node dissection. M, mucosal; SM, submucosal. Deaths other than gastric carcinoma were censored at the time of death. $P = 0.013$.

### *Univariate and multivariate analysis*

Table IV shows the results of univariate analysis of the ten factors examined as potential prognostic indicators. Univariate analysis indicated that nodal involvement, nodal dissection and depth of invasion were significantly related to outcome ($P < 0.0001$, $P < 0.005$ and $P < 0.01$ respectively). Multivariate analysis (Table V) revealed that the factor most highly correlated with outcome was nodal involvement (risk ratio: 8.4; $P < 0.0001$), followed by node dissection (risk ratio: 5.8; $P < 0.005$). Multivariate analysis indicated that the depth of invasion and macroscopic type were not significantly related to outcome.

### Discussion

Until now it has not been determined whether limited or aggressive lymph node dissection should be employed for EGC. To our knowledge there is only one study (Kodama *et al.*, 1981) in which limited or aggressive lymph node dissection for EGC was compared. In that study, the 5 year survival rate of patients with extensive lymph node dissection did not differ from those with simple resection, but the sample size was not as large.

In the present study, 392 patients with EGC were evaluated and followed up 10 years after surgery. The reason why we followed for such a long time is that cancer death occurs often 5–10 years after surgery (Itoh *et al.*, 1989; Furusawa *et al.*, 1991; Moreaux and Bougaran, 1993; Sano *et al.*, 1993). Indeed, the present study revealed that only 43% of the recurred patients died 5 years after surgery. The 10 year survival rate of patients wtih ELND gastrectomy was significantly higher than that of patients with LLND gastrectomy. This difference was observed only in patients with nodal metastasis, and not in those without metastasis. Thus ELND gives a survival benefit to node-positive EGC patients.

When more extensive lymph node dissection is performed, the capacity to stage patients by nodal status is enhanced. The impact of screening programmes on clinical presentation is a fact which has been reported in other cancers as well. Stage shifting must be considered in interpreting the present results. We conducted a thorough pathological examination of all lymph nodes in the resected specimens in the two groups, and found that in the extensive lymphadenectomy group, not only was the total number of dissected nodes increased, but also the number of metastatic nodes was increased by the ELND procedure. However, no increment in the incidence of patients with positive nodes in the ELND group was observed compared with the LLND group. The extended procedure did not result in any significant stage shifting in these patients.

There are some reports that EGC patients with nodal metastasis have a poorer prognosis than those with cancer-negative nodes (Habu *et al.*, 1986; Marczell *et al.*, 1988; Inoue *et al.*, 1991; Lawrence and Shiu, 1991; Santoro *et al.*, 1991; Guadagni *et al.*, 1993; Pinto *et al.*, 1994). This aspect is supported by our findings not only for the LLND group but also for the ELND group. Although ELND brought about a significantly increased number of positive lymph nodes, the survival rate of patients with nodal metastasis in the ELND group was significantly increased from that in the LLND group. These observations suggest that most of the EGC

**Table IV** Results of univariate analysis of relationship of clinical and pathological variables to disease-specific survival rate in patients with early gastric carcinoma

| *Characteristic* | *Number of patients* | *10 year survival rate (%)*[a] | *95% confidence interval* | P[b] |
|---|---|---|---|---|
| Age (years) | | | | 0.87 |
| <59 | 197 | 94.4 | 90.9–97.9 | |
| ≥59 | 195 | 94.8 | 91.5–98.1 | |
| Sex | | | | 0.89 |
| Female | 121 | 93.9 | 88.5–99.3 | |
| Male | 271 | 95.7 | 92.9–98.5 | |
| Nodal dissection | | | | <0.005 |
| LLND | 97 | 88.1 | 80.7–95.5 | |
| ELND | 295 | 97.9 | 96.1–99.7 | |
| Reconstruction | | | | 0.21 |
| Billroth I | 336 | 96.3 | 94.0–98.6 | |
| Billroth II | 56 | 92.0 | 93.1–100 | |
| Location of tumour | | | | 0.80 |
| Distal | 210 | 94.9 | 91.3–98.5 | |
| Middle | 182 | 95.8 | 92.5–99.1 | |
| Macroscopic type | | | | 0.063 |
| Non-ulcerated | 126 | 93.0 | 87.8–98.2 | |
| Ulcerated | 266 | 96.2 | 93.9–99.1 | |
| Tumour size (cm) | | | | 0.71 |
| ≥3.0 | 196 | 93.0 | 87.8–98.2 | |
| <3.0 | 196 | 96.2 | 93.9–99.1 | |
| Depth of invasion | | | | <0.01 |
| Submucosal | 182 | 91.6 | 86.9–96.3 | |
| Mucosal | 210 | 98.5 | 96.4–100 | |
| Nodal involvement | | | | <0.0001 |
| Present | 51 | 77.4 | 64.9–89.9 | |
| Absent | 341 | 98.0 | 96.0–100 | |
| Number of positive nodes | | | | 0.80 |
| One | 21 | 78.3 | 59.4–97 | |
| Two or more | 30 | 82.0 | 67.7–96.3 | |
| Histological type | | | | 0.17 |
| Differentiated | 271 | 93.9 | 90.4–97.4 | |
| Undifferentiated | 121 | 97.6 | 94.3–100 | |

ELND, extensive lymph node dissection; LLND, limited lymph node dissection. [a]Deaths other than gastric carcinoma were censored at the time of death. [b]Log-rank test.

**Table V** Multivariate analysis of prognostic factors for 10 year disease-specific survival

| *Characteristic* | P | *Risk ratio* | *95% confidence interval* |
|---|---|---|---|
| Nodal involvement | | | |
| Present/absent | <0.0001 | 8.4 | 2.9–24.9 |
| Node dissection | | | |
| LLND/ELND | <0.005 | 5.8 | 2.0–16.6 |
| Macroscopic type | | | |
| Non-ulcerated/ulcerated | 0.51 | | |
| Depth of invasion | | | |
| Submucosal/mucosal | 0.21 | | |

ELND, extensive lymph node dissection; LLND, limited lymph node dissection. Deaths other than gastric carcinoma were censored at the time of death. Cox proportional hazards model.

patients with nodal metastasis can be cured by ELND and that nodal metastasis does not indicate systemic disease.

When the lymph nodes are enlarged, metastasis can generally be easily diagnosed by gross examination. However, when they are not enlarged, surgeons cannot easily determine intraoperatively whether metastatic foci are present or not. The nodal status of EGC are generally of the latter type (Sano *et al.*, 1994). Although frozen lymph node sections certainly provide reliable information, the ability to examine a large number of lymph nodes is limited. Thus, the nodal status cannot be accurately diagnosed before the postoperative pathological examination.

Some reports indicate that the survival rate differs according to whether the depth of invasion is mucosal or submucosal (Gentsch *et al.*, 1981; Takagi *et al.*, 1987; Craanen *et al.*, 1991; Lawrence and Shiu, 1991; Inoue *et al.*, 1991; Guadagni *et al.*, 1993; Moreaux and Bougaran, 1993). It is well established that, as the depth of gastric carcinoma invasion into the gastric wall increases, the frequency and the degree of nodal metastasis also increases. Lymph nodal metastasis is present in 1.8–4.8% of EGC patients with mucosal invasion, but in 16.7–23.8% of those with submucosal invasion (Habu *et al.*, 1986; Korenaga *et al.*, 1986; Ohta *et al.*, 1987; Iriyama *et al.*, 1989; Sowa *et al.*, 1989; Inoue *et al.*, 1991; Moreaux and Bougaran, 1993). Thus, the local invasiveness of EGC may be correlated with the survival rate (Lawrence and Shiu, 1991). However, in the present study, the survival of patients with submucosal invasion who underwent the ELND procedure was significantly higher than those who underwent the LLND procedure, and almost similar to that of patients with mucosal invasion who underwent either procedure. These findings indicate that the ELND procedure should be used in patients with submucosal carcinoma.

The reported incidence of nodal metastasis was 12.9% among 791 patients with EGC treated at the Cancer Institute Hospital, Tokyo (Takagi *et al.*, 1987); 8.8% in $n_1(+)$, 3.2% in $n_2(+)$ and 0.5% in $n_3(+)$. Our study demonstrated almost the same incidence of metastasis, 8.5% in $n_1(+)$, 3.1% in $n_2(+)$ and 0.6% in $n_3(+)$. When dealing with small number of patients, the low incidence in either $n_2(+)$ or $n_3(+)$ may be insignificant, a fact that could mask the superiority of ELND for EGC with nodal metastasis.

In contrast, it is debatable whether aggressive surgery should be considered for mucosal carcinomas. Lymph node metastases do occur in patients once the basement membrane has been breached since there are lymphatics within the deeper layers of the mucosa, above the submucosa. Korenaga *et al.* (1986) recommended lymph node dissection even in patients with mucosal carcinoma, because they found metastases to distant lymph nodes in three of 568 patients with mucosal EGC. Lawrence and Shiu (1991) also emphasised that extended lymphadenectomy should be used in all patients with EGC, even in those in whom mucosal invasion is identified. However, in our study, the survival rate for patients with mucosal carcinoma treated using the ELND procedure was only slightly different from those treated using the LLND procedure. Numerous clinicopathological studies have demonstrated that the frequency of nodal metastasis in EGC with mucosal invasion is less than 5%. The degree of nodal invasion is almost always limited to $n_1(+)$, and in less than 1% of the cases with $n_2(+)$ (Habu *et al.*, 1986; Korenaga *et al.*, 1986). EGC patients with $n_2(+)$ would have been cured only by the ELND procedure but this would have resulted in a comparatively small improvement in the survival rate. This relatively small survival benefit must be considered against the increased operative risks of extended lymphadenectomy. Although the operative mortality rate of 2% associated with the ELND procedure seems to be acceptable, some researchers have reported that more aggressive surgery may be related to higher operative morbidity and mortality (Diggory and Cuschieri, 1985; Heberer *et al.*, 1988; Dent *et al.*, 1988). In addition, the ELND procedure presents the disadvantage of longer operation time and greater blood loss, although these factors were not associated with any increase in the mortality rate (de Aretxabala *et al.*, 1987; Smith *et al.*, 1991; Pacelli *et al.*, 1993). Therefore, the most appropriate treatment in an individual patient with EGC in whom invasion is limited to the mucosal layer must be determined after full consideration of all the risks and benefits. Recently, in some selected cases of gastric mucosal carcinoma, Japanese surgeons attempted cautiously to perform endoscopic mucosal resection or limited surgery such as local resection, segmental resection, pylorus-preserving gastrectomy and $R_1$ gastrectomy. Certainly, such limited procedures improved patients' quality of life, but the value of limited procedures should be estimated inclusive of the risk of carcinoma recurrence in future.

The distinction of mucosal carcinoma from submucosal carcinoma is important in limited procedures. Radiographic or endoscopic diagnosis is often inaccurate. It is not rare for a tumour that has been grossly diagnosed as mucosal carcinoma to infiltrate into the submucosal layer or deeper. We know for certain that the protruded type EGC less than 2.0 cm in diameter is limited to the mucosa and that it lacks nodal metastasis (Kitaoka *et al.*, 1984; Korenaga *et al.*, 1986; Ohta *et al.*, 1987; Iriyama *et al.*, 1989). In addition, if a tumour shows a depressed differentiated adenocarcinoma less than 1.0 cm in diameter, then it has little chance of nodal metastasis (Takekoshi *et al.*, 1994). A limited procedure can be indicated in these cases only. Otherwise, endoscopic ultrasonography can be a useful tool for deciding the indication of limited procedures. Yoshino *et al.* (1987) reported that the rate of correct diagnosis in endoscopic ultrasonography was 83.3% in mucosal carcinoma and 71.4% in carcinoma with submucosal invasion, while the accurate rate of radiographic examination was 73.0% and 64.7% respectively. The accuracy of endoscopic ultrasonography in our own experience was 88.9% in mucosal carcinoma and 62.5% in submucosal carcinoma. Therefore the accuracy rate of endoscopic ultrasonography is higher than that of radiographic examination, but it is not high enough to be used to indicate a limited procedure. Further development of instruments for endoscopic ultrasonography may facilitate more accurate distinction between these two depths of carcinoma invasion.

In conclusion, our retrospective findings suggest that aggressive lymph node dissection, in spite of the prolonged operation time and increased loss of blood, increases the survival rate of EGC patients with nodal metastasis. On the other hand, EGC without nodal involvement does not require any aggressive nodal dissection. We need a search for a new diagnostic technique to differentiate EGC patients regarding the presence or absence of nodal metastasis preoperatively or perioperatively.

**Acknowledgements**

We are grateful to Professor K Saijyo (Department of Hygiene, School of Medicine, Kanazawa University) for his advice in statistical analysis.

**References**

COX DR. (1972). Regression models and life table. *J.R. Stat. Soc. Series B*, **34**, 187–220.

CRAANEN ME, DEKKER W, FERWERDA J, BLOK P AND TYTGAT GNJ. (1991). Early gastric cancer: a clinicopathologic study. *J. Clin. Gastroenterol.*, **13**, 274–283.

CUSCHIERI A. (1986). Gastrectomy for gastric cancer: definitions and objectives. *Br. J. Surg.*, **73**, 513–514.

DE ARETXABALA X, KONISHI K, YONEMURA Y, UENO K, YAGI M, NOGUCHI M, MIWA K AND MIYAZAKI I. (1987). Node dissection in gastric cancer. *Br. J. Surg.*, **74**, 770–773.

DENT DM, MADDEN MV AND PRICE SK. (1988). Randomised comparison of $R_1$ and $R_2$ gastrectomy for gastric carcinoma. *Br. J. Surg.*, **75**, 110–112.

DIGGORY RT AND CUSCHIERI A. (1985). R2/3 gastrectomy for gastric carcinoma: an audited experience of a consecutive series. *Br. J. Surg.*, **72**, 146–148.

FARLEY DR, DONOHUE JH, NAGORNEY DM, CARPENTER HA, KATZMANN JA AND ILSTRUP DM. (1992). Early gastric cancer. *Br. J. Surg.*, **79**, 539–542.

FURUSAWA M, NOTSUKA T AND TOMODA H. (1991). Recurrence of early gastric cancer. *Semin. Surg. Oncol.*, **7**, 344–350.

GENTSCH HH, GROITL H AND GIEDL J. (1981). Results of surgical treatment of early gastric cancer in 113 patients. *World J. Surg.*, **5**, 103–107.

GUADAGNI S, REED PI, JOHNSTON BJ, DE-BERNARDINIS G, CATARCI M, VALENTI M, DI-ORIO F AND CARBONI M. (1993). Early gastric cancer: follow-up after gastrectomy in 159 patients. *Br. J. Surg.*, **80**, 325–328.

HABU H, TAKESHITA K, SUNAGAWA M AND ENDO M. (1986). Lymph node metastasis in early gastric cancer. *Int. Surg.*, **71**, 244–247.

HARAGUCHI M, KORENAGA D, OKAMURA T, TSUJITANI S AND SUGIMACHI K. (1990). A small early carcinoma of the stomach with extra-perigastric lymph-node metastasis. *Jpn. J. Surg.*, **20**, 111–114.

HEBERER G, TEICHMAN RK, KRÄMLING HJ AND GÜNTHER B. (1988). Results of resection for carcinoma of the stomach: the European experience. *World J. Surg.*, **12**, 374–381.

HEESAKKERS JP, GOUMA DJ, THUNNISSEN FB, BEMELMANS MH AND VON MEYENFELDT MF. (1994). Non-radical therapy for early gastric cancer. *Br. J. Surg.*, **81**, 551–553.

INOUE K, TOBE T, KAN N, NIO Y, SAKAI M, TAKEUCHI E AND SUGIYAMA T. (1991). Problems in the definition and treatment of early gastric cancer. *Br. J. Surg.*, **78**, 818–821.

IRIYAMA K, ASAKAWA T, KOIKE H, NISHIWAKI H AND SUZUKI H. (1989). Is extensive lymphadenectomy necessary for surgical treatment of intramucosal carcinoma of the stomach? *Arch. Surg.*, **124**, 309–311.

IRVIN TT AND BRIDGER JE. (1988). Gastric cancer: an audit of 122 consecutive cases and results of $R_1$ gastrectomy. *Br. J. Surg.*, **75**, 106–109.

ITOH H, OOHATA Y, NAKAMURA K, NAGATA T, MIBU R AND NAKAYAMA F. (1989). Complete ten-year postgastrectomy follow-up of early gastric cancer. *Am. J. Surg.*, **158**, 14–16.

JAPANESE RESEARCH SOCIETY FOR GASTRIC CANCER. (1981). The general rules for the gastric cancer study in surgery and pathology. Part I. Clinical classification. Part II. Histological classification of gastric cancer. *Jpn. J. Surg.*, **11**, 127–145.

KAPLAN EL AND MEIER P. (1958). Nonparametric estimation for incomplete observations. *J. Am. Stat. Assoc.*, **53**, 457–481.

KITAOKA K, YOSHIKAWA K, HIROTA T AND ITABASHI M. (1984). Surgical treatment of early gastric cancer. *Jpn. J. Clin. Oncol.*, **14**, 283–293.

KODAMA Y, SUGIMACHI K, SOEJIMA K, MATSUSAKA T AND INOKUCHI K. (1981). Evaluation of extensive lymph node dissection for carcinoma of the stomach. *World J. Surg.*, **5**, 241–248.

KORENAGA D, HARAGUCHI M, TSUJITANI D, OKAMURA T, TAMADA T AND SUGIMACHI K. (1986). Clinicopathological features of mucosal carcinoma of the stomach with lymph node metastasis in eleven patients. *Br. J. Surg.*, **73**, 431–433.

LAWRENCE M AND SHIU MH. (1991). Early gastric cancer. Twenty-eight-year experience. *Ann. Surg.*, **213**, 327–334.

LEHNERT T, STERNBERG SS, SPROSSMANN M AND DECOSSE JJ. (1989). Early gastric cancer. *Am. J. Surg.*, **157**, 202–207.

MAEHARA Y, OKUYAMA T, OSHIRO T, BABA H, ANAI H, AKAZAWA K AND SUGIMACHI K. (1993). Early carcinoma of the stomach. *Surg. Gynecol. Obstet.*, **177**, 593–597.

MARCZELL AP, ROSEN HR AND HENTSCHEL E. (1988). Diagnosis and tactical approach to surgery for early gastric carcinoma: a retrospective analysis of the past 16 years in an Austrian general hospital. *Gastroenterol. Jpn.*, **24**, 732–736.

MARUYAMA K, OKABAYASHI K AND KINOSHITA T. (1987). Progress in gastric cancer surgery in Japan and its limits of radicality. *World J. Surg.*, **11**, 418–425.

MOREAUX J AND BOUGARAN J. (1993). Early gastric cancer: a 25 year surgical experience. *Ann. Surg.*, **217**, 347–355.

OHTA H, NOGUCHI Y, TAKAGI K, NISHI M, KAJITANI T AND KATO Y. (1987). Early gastric carcinoma with special reference to macroscopic classification. *Cancer*, **60**, 1099–1106.

PACELLI F, DOGLIETTO GB, BELLANTONE R, ALFIERI S, SGADARI A AND CRUCITTI F. (1993). Extensive versus limited lymph node dissection for gastric cancer: a comparative study of 320 patients. *Br. J. Surg.*, **80**, 1153–1156.

PERCIVALE P, BERTOGLIO S, MUGGIANU M, ASTE H, SECCO GB, MARTINES H, MORESCO L AND CAFIERO F. (1989). Long-term postoperative results in 54 cases of early gastric cancer: the choice of surgical procedure. *Eur. J. Surg. Oncol.*, **15**, 436–440.

PETO R AND PIKE MC. (1973). Conservatism in the approximation $(O\text{-}E)^2/E$ in the log-rank test for survival data or tumour incidence data. *Biometrics*, **29**, 579–584.

PINTO E, ROVIELLO F, DE STEFANO A AND VINDIGNI C. (1994). Early gastric cancer: report on 142 patients observed over 13 years. *Jpn. J. Clin. Oncol.*, **24**, 12–19.

SANO T, SASAKO M, KINOSHITA T AND MARUYAMA K. (1993). Recurrence of early gastric cancer: follow-up of 1475 patients and review of the Japanese literature. *Cancer*, **72**, 3174–3178.

SANO T, KOBORI O, NAGAWA H AND MUTO T. (1994). The macroscopic diagnosis of lymph node metastasis from early gastric cancer. *Surg. Today*, **24**, 37–39.

SANTORO E, GAROFALO F, SCUTARI F, ZANARINI T, CARLINI M AND SANTORO Jr E. (1991). Early gastric cancer: total gastrectomy *vs.* distal resection: results of a study of 271 cases. *Hepato-Gastroenterol.*, **38**, 421–429.

SMITH JW, SHIU MH, KELSEY L, MURRAY F AND BRENNAN F. (1991). Morbidity of radical lymphadenectomy in the curative resection of gastric carcinoma. *Arch. Surg.*, **126**, 1469–1473.

SOWA M, KATO Y, NISHIMURA M, KUBO T, MACAW H AND UMEYAMA K. (1989). Surgical approach to early gastric cancer with lymph node metastasis. *World J. Surg.*, **13**, 630–636.

TAKAGI K, NISHI M AND KAJITANI T. (1987). Surgical treatment of gastric cancer today. *Wien Klin. Wochenschr.*, **99**, 410–415.

TAKEKOSHI T, BABA Y, OTA H, KATO Y, YANAGISAWA A, TAKAGI K AND NOGUCHI Y. (1994). Endoscopic resection of early gastric carcinoma: results of a retrospective analysis of 308 cases. *Endoscopy*, **26**, 352–358.

YOSHINO J, NAKAZAWA S, NAKAMURA T, YAMANAKA T, HASE T AND KOJIMA Y. (1987). The depth of invasion of gastric cancer estimated by roentgenography and endoscopic ultrasonography. *Stom. Intest. (I-to-Cho)*, **22**, 169–177.